# Aquatic Resistome in Freshwater and Marine Environments: Interactions Between Commensal and Pathogenic in the Context of Aquaculture and One Health

**DOI:** 10.3390/microorganisms13071591

**Published:** 2025-07-06

**Authors:** Ana V. Mourão, Diana Fernandes, Telma de Sousa, Rita Calouro, Sónia Saraiva, Gilberto Igrejas, Patrícia Poeta

**Affiliations:** 1 MicroART-Antibiotic Resistance Team, Department of Veterinary Sciences, University of Trás-os-Montes and Alto Douro, 5000-801 Vila Real, Portugal; vanessamourao123@gmail.com (A.V.M.); dianaivfernandes@gmail.com (D.F.); telmaslsousa@hotmail.com (T.d.S.); ritasousacalouro@gmail.com (R.C.); soniasarai@gmail.com (S.S.); 2 Department of Genetics and Biotechnology, University of Trás-os-Montes and Alto Douro, 5000-801 Vila Real, Portugal; gigrejas@utad.pt; 3 Functional Genomics and Proteomics Unit, University of Trás-os-Montes and Alto Douro, 5000-801 Vila Real, Portugal; 4 Associated Laboratory for Green Chemistry, University NOVA of Lisbon, 1099-085 Caparica, Portugal; 5 CECAV—Veterinary and Animal Research Center, Universidade de Trás-os-Montes and Alto Douro, 5000-801 Vila Real, Portugal; 6 Polytechnic Institute of Santarém, School of Agriculture, Quinta do Galinheiro, 2001-904 Santarém, Portugal; 7 Veterinary and Animal Research Centre, Associate Laboratory for Animal and Veterinary Science (AL4AnImalS), University of Trás-os-Montes and Alto Douro, 5000-801 Vila Real, Portugal

**Keywords:** aquatic resistomes, horizontal gene transfer, antimicrobial resistance, mobile genetic elements, One Health, metagenomics

## Abstract

Aquatic resistomes are important reservoirs of antibiotic resistance genes (ARGs) and their precursors, which can proliferate and dissipate in pathogenic microorganisms that affect humans and animals, especially due to anthropogenic pressures such as the intensive use of antibiotics in aquaculture, often without effective regulation. This review addresses the mechanisms of horizontal gene transfer (HGT) in the dissemination of ARGs through mobile genetic elements (MGEs). In freshwater, genera such as *Aeromonas*, *Pseudomonas* and *Microcystis* stand out as vectors of ARGs. In the context of One Health, it is essential to implement sound public policies and strict regulations on the use of antibiotics in aquaculture, and the use of monitoring tools such as environmental DNA (eDNA) and metagenomics allows for the early detection of ARGs, contributing to the protection of human, animal and environmental health.

## 1. Introduction

Antimicrobial resistance (AMR) refers to the ability of bacteria to survive exposure to antibiotics that were previously effective against them. This resistance can be intrinsic, acquired or adaptative and may lead to the emergence of resistance bacterial clones capable of withstanding a wide range of antibiotic levels, depending on the resistance mechanism involved [1]. Although some resistance mechanisms occur naturally, it is the selective pressure from antibiotic use, driven largely by human practices, that plays a key role in the development and dissemination of antimicrobial resistance [2]. The consequences are significant, as AMR compromises the effectiveness of standard treatments, leading to prolonged illness, increased healthcare costs, and higher mortality. According to the World Health Organization (WHO), the emergence and spread of AMR in human pathogens is a global threat that increasingly undermines the successful treatment of infectious diseases [1].

AMR is a complex issue that extends beyond human health, involving animals, the environment and agricultural practices [1,3]. The One Health concept provides an integrated framework that acknowledges the interconnectedness of human, animal and environmental health. It emphasizes the need for cross-disciplinary collaboration to effectively address health threats like AMR, recognizing that the emergence and spread of resistance can occur across all sectors and affect all living beings and ecosystems [3].

Given the interconnectedness of human, animal, and environmental health, the spread of AMR in aquatic ecosystems exemplifies how the One Health approach is crucial in addressing this global issue [4]. Aquatic ecosystems, including freshwater and marine environments, are essential for biodiversity and food security. However, they are increasingly threatened by the spread of AMR. Aquaculture plays a significant role in this issue, as antibiotics are often used in aquatic habitats, either directly or through animal feed, to manage infections. This can lead to the emergence of resistant bacteria, which are then released into the environment, contributing to the spread of AMR. The spread of AMR in aquatic ecosystems poses risks to both public health and ecosystem stability [5].

## 2. The Aquatic Resistome: Concepts and Mechanisms

Aquatic resistomes comprise a set of antibiotic resistance genes (ARGs), such as *bla_TEM_* and *mecA*, and their precursors in pathogens, antibiotic producers and benign environmental bacteria present in the microbial communities of freshwater and marine aquatic environments, which can proliferate and dissipate into pathogenic microorganisms affecting humans and animals, threatening the efficacy of antimicrobial therapies and constituting a public health risk [6,7,8].

Resistomes can be classified into native (or intrinsic) resistomes and acquired resistomes (Table 1) [9].

Unlike VGT, which occurs from parent to offspring, HGT allows the direct transfer of genetic material between phylogenetically distant bacterial species, favoring the rapid spread of ARGs in aquatic ecosystems where there is high microbial density and diversity [15].

The main HGT mechanisms include conjugation, transformation and transduction. Conjugation is an effective process for spreading ARGs in aquatic environments, especially in biofilms, as it involves the transfer of genetic material between two bacterial cells (a donor cell to a recipient cell) through direct physical contact, which is facilitated by specialized structures such as conjugation pili, Transformation refers to the process by which extracellular DNA from lysed donor bacteria is incorporated by recipient bacteria into their genomes, allowing the recipient bacteria to acquire new attributes. Finally, transduction occurs when a bacteriophage accidentally transfers ARGs by encapsulating fragments of bacterial DNA with ARGs from the donor bacteria to the recipient bacteria so that the recipient bacteria can acquire new characteristics [16,17,18,19].

**Figure 1:**
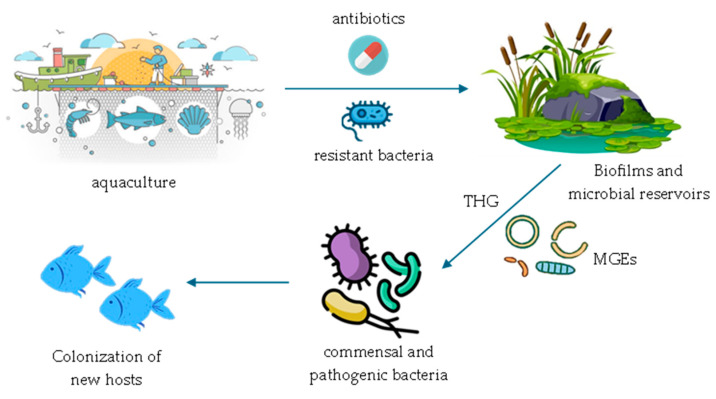
Transmission of ARGs in aquatic environments.

The acquisition and spread of ARGs in aquatic environments are often facilitated by mobile genetic elements (MGEs), such as plasmids, transposons and integrons, which are sequences of genetic material capable of moving within the genome or between different genomes (Table 2) (Figure 1) [20,21].

Several environmental factors favor the mobility and persistence of ARGs in the aquatic resistome, such as recombination hotspots (sites such as sediments and biofilms that act as vital reservoirs for ARGs and are characterized by high microbial density and inter- and intra-species genetic exchange) and co-selection by heavy metals and organic contaminants (some MGEs contain genes that confer simultaneous resistance to antibiotics, heavy metals such as copper, zinc, and mercury, and organic contaminants, including pesticides and biocides). For example, studies have shown that *Aeromonas salmonicida* isolates from aquaculture environments carry plasmids encoding both mercury resistance operons and antibiotic resistance genes such as *sulII*. This is an example of co-resistance, where genes conferring resistance to heavy metals and antibiotics are located on the same mobile genetic elements [16,24,25,26].

## 3. Commensal and Pathogenic Bacteria in Freshwater

Commensal bacteria are naturally occurring microorganisms that make up the native microbiota of aquatic environments, including both water columns and sediments. Although typically benign, these bacteria play a key ecological role by contributing to the balance and functioning of microbial communities. They can act as a natural reservoir of ARGs. This silent reservoir function highlights the relevance of commensal bacteria in the emergence and spread of antimicrobial resistance in freshwater ecosystems [27].

Freshwater environments are particularly relevant to the spread of pathogens due to their essential role in the survival of numerous organisms, the presence of aquatic stages in the life cycles of many vectors and intermediate hosts, and the natural convergence of a wide range of species, both aquatic and terrestrial, around these habitats. Additionally, the frequent human-induced disturbances in freshwater systems can alter ecological interactions and influence disease dynamics, making these ecosystems important sites for the emergence and transmission of infectious agents [28].

As highlighted in recent studies, *Aeromonas*, *Pseudomonas* and *Vibrio* are major pathogens in aquaculture. These bacteria are known to infect a wide range of fish species, causing septicemic infections, and they are associated with gastrointestinal and other infections in humans [29]. Moreover, these pathogens are capable of colonizing water–soil transition zones, areas where water meets the sediment. These transitional zones provide an ideal environment for the persistence of these bacteria, facilitating their interaction with both aquatic and soil microbial communities, which can contribute to the spread of antimicrobial resistance and increase the risk of infections in aquatic ecosystems [30].

For example, *Aeromonas hydrophila*, although considered a rare human pathogen, has been implicated in soft tissue infections following freshwater-related injuries. One case involved a previously healthy 13-year-old girl who developed severe septic arthritis of the knee after a traumatic injury in a private freshwater lake. This case highlights the pathogen’s ability to cause rapid infections in joints, particularly following untreated freshwater. Additionally, *A. Hydrophila* has shown persistence even after chlorination, underscoring its resilience in recreational freshwater environments and the need for improved disinfection strategies [31].

A rare but fatal case of community-acquired pneumonia caused by *Pseudomonas aeruginosa* was reported in a previously healthy 49-year-old woman after using a hotel hot tub during a spa holiday. Despite receiving appropriate antimicrobial treatment, the patient died of septic multiorgan failure. Environmental sampling revealed extremely high levels of *P. aeruginosa* in the hot tub water, and molecular typing confirmed the genetic similarity between environmental and clinical isolates, suggesting the infection originated from a poorly maintained water system with biofilm formation [32].

Septic arthritis caused by *Aeromonas*, for example, had only nine documented cases in the English literature until 2011, while severe community-acquired pneumonias caused by *Pseudomonas* are often linked to specific exposures, such as contaminated water in recreational equipment [31]. Despite their low incidence, their relevance for environmental surveillance is significant, as they act as sentinel events. They reveal, for instance, how environmental bacteria like *Aeromonas* persist in chlorinated water due to adaptation to disinfectants [33] or how *Pseudomonas* forms resistant biofilms in poorly maintained water systems, where exposure to antibiotic residues or heavy metals can select for multidrug-resistant strains [32]. Genomic studies (using PFGE/MLST) further confirm the identity between clinical and environmental isolates in these contexts, confirming direct transmission from the environment to humans. However, traditional AMR environmental surveillance systems often overlook non-enteric pathogens like *Aeromonas* and *Pseudomonas*, prioritizing bacteria such as *E. coli* or *Salmonella* [33]. This gap is concerning because such cases demonstrate that (1) *Aeromonas* can cause invasive infections in vulnerable groups (e.g., immunosuppressed children), (2) community-acquired *Pseudomonas* infections have high lethality (over 10% in bacteremia pneumonia) [34,35], and (3) recreational or natural aquatic environments are underestimated reservoirs of AMR. The detection of resistance to β-lactams and fluoroquinolones in *Aeromonas* from water sources, or multidrug resistance in *Pseudomonas* from hot tubs, reflects environmental selective pressures that monitoring programs should incorporate [36]. Thus, although these cases are epidemiologically uncommon, their integrated clinical–environmental analysis is crucial to guide policies such as regulation of disinfection in recreational waters and biofilm monitoring, identify risks to vulnerable populations, and broaden the scope of environmental surveillance beyond enteric pathogens. Ignoring such examples may compromise early detection of emerging AMR threats in non-hospital ecosystems [37].

Bacterial evolution and adaptation are closely linked to environmental conditions, with biofilm formation being a key strategy for survival in aquatic habitats. These biofilms, often multispecies and associated with suspended organic particles, provide a microenvironment where close physical proximity between cells facilitates HGT [31,32,38,39].

## 4. Bacteria and Antibiotic Resistance

Environmental bacteria, such as cyanobacteria, exhibit remarkable genomic plasticity, with genome sizes ranging from 1.44 Mb to over 9 Mb and, in some cases, such as *Synechocystis* PCC 6803, displaying extreme polyploidy with up to 218 chromosome copies per cell. These features may contribute to their ability to harbor ancient ARGs, retained though evolutionary timescales. Moreover, the presence of diverse regulatory elements, including two-component systems and regulatory RNAs, highlights the adaptive potential of these organisms to environmental stressors, including anthropogenic pollutants and antibiotic exposure [40]. Interestingly, they also produce secondary metabolites, which may influence the expression of ARGs in other bacterial species, affecting the broader spread of resistance in these environments [41].

Recent studies have highlighted the presence of a wide variety of ARGs in aquatic environments, including those associated with β-lactamases, tetracycline and macrolides in bacterial genera such as *Microcystis*. A study conducted in Lake Taihu identified a high diversity of ARGs during cyanobacterial blooms, particularly in *Microcystis* populations. Using 16 rRNA sequencing and qPCR, researchers detected over 120 ARGs across several antibiotic classes, including β-lactams, tetracyclines and macrolides (36). In addition to harboring their own ARGs, *Microcystis aeruginosa* may also acquire resistance through interactions with surrounding microbial communities. Another recent study demonstrated that xenic strains of *M. aeruginosa* exhibited resistance to β-lactam antibiotics, unlike axenic strains, due to the presence of β-lactamase-producing bacteria in their phycosphere. Metagenomic analysis revealed the dominance of *bla_OXA_* and other class A β-lactamase genes (*AST-1*, *FAR-1*) in the xenic cultures. These findings highlight Microcystis as important reservoirs of ARGs in freshwater systems, raising concerns about their role in the environmental dissemination of antimicrobial resistance [42].

In addition to carrying ARGs, cyanobacterial communities are often embedded in microbial assemblages where HGT plays a key role in the dissemination of resistance (36). One of the main mechanisms involved in this process is the presence of MGEs such as class 1 integrons (intl1), which can capture and disseminate resistance cassettes among diverse bacterial populations. These elements are particularly prevalent in environments exposed to anthropogenic pressures. For example, Koczura et al. (2022) reported significantly higher abundances of *intl1*, *sul1* and *sul2* genes in river water and sediments impacted by urban and agricultural effluents compared to less affected sites [43]. These findings underscore the influence of anthropogenic activity on the enrichment of resistance determinants in freshwater ecosystems.

Beyond MGEs, cell lysis plays a significant role in the release of genetic material such as extracellular DNA (eDNA) into aquatic environments, particularly under stress conditions. This genetic material can serve not only as a structural component of biofilms but also as a reservoir of genes potentially available for HGT, promoting the dissemination of genetic traits including ARGs in aquatic ecosystems [44].

In aquatic environments, cyanobacteria often form close associations with heterotrophic bacteria within mixed-species biofilms. These consortia are stabilized by the excretion of organic carbon compounds by cyanobacteria, which support the growth of heterotrophic populations. Such structural and metabolic interactions promote cell proximity and communication, creating favorable conditions for HGT. These mixed biofilms thus represent potential hotspots for the exchange of genetic material, including ARGs, between phylogenetically distinct bacteria [45].

## 5. Aquaculture as a Vector of Dissemination

In aquaculture, the use of antibiotics is a common practice both for therapeutic purposes, aimed at treating bacterial infections in fish and shellfish, and for prophylactic purposes, with the aim of preventing disease outbreaks in intensive production systems, where the risk of pathogenic outbreaks is high due to high population density and animal stress. However, in many countries without strict regulations, antibiotics are administered without proper veterinary supervision and often without the necessary control over dosages and duration of the procedure, which contributes significantly to the development of resistance [46,47]. In addition, this uncontrolled use results in the accumulation of antibiotic residues both in the water column and in aquatic sediments, favoring the spread of ARGs [48].

Among the antibiotic-resistant bacteria frequently associated with aquaculture, genera such as Aeromonas, Vibrio, Pseudomonas and *Escherichia coli* stand out. A study conducted in Vietnam by Nguyen et al. (2014) analyzed 208 bacterial isolates from intensive catfish farming systems in the Mekong Delta, of which 116 were *Pseudomonas* and 92 *Aeromonas* [49].

In studies carried out by Salgueiro et al. (2024), antibiotic resistance profiles were identified in sediments from aquacultures in Portuguese estuaries in the Sado and Lima rivers and the Ria de Aveiro, showing a significant increase in ARGs in aquatic environments adjacent to fish farms [50].

Aquaculture production sites, especially intensive and semi-intensive fish farming areas, have shown a high proliferation of multidrug-resistant bacteria due to the high density of organisms and frequent use of antibiotics that favor the spread of ARGs [51]. In this context, the study by Saqr et al. (2016), showed that 95 per cent of *Escherichia coli* isolates from Nile tilapia (Oreochromis niloticus) showed multiple resistance to classes of antibiotics, including tetracyclines, ampicillins (AML), streptomycins (S) and sulfamethoxazole–trimethoprim (SXT) and tetracycline (TE), highlighting the selective pressure exerted by intensive aquaculture practices [52].

The discharge of untreated effluent from fish farming into the aquatic environment and cross-contamination between production batches due to inadequate biosecurity and hygiene practices favor the spread of ARGs by introducing resistant bacteria, residual antibiotics and MGEs that favor HGT [40,53,54,55].

## 6. One Health Implications and Future Prospects

In the context of AMR, One Health recognizes that human, animal and environmental health are interconnected and share an ecosystem where ARGs and multidrug-resistant bacteria circulate continuously along the trophic chain and through water, soil and food, which highlights the need for integrated surveillance and data sharing between all health sectors to monitor AMR, identify emerging patterns of resistance and locate sources of contamination [56].

In recent years, AMR monitoring tools such as eDNA and metagenomics have enabled more sensitive and rapid detection of ARGs in water samples. eDNA analysis allows the extraction and analysis of genetic material obtained directly from environmental samples, without the need to capture the organisms, thus enabling the rapid detection of ARGs. eDNA offers high sensitivity, low cost, and speed but is limited to identifying previously known genes and does not provide genetic context. On the other hand, metagenomics is based on second generation (such as Illumina) and third generation (such as Oxford Nanopore or PacBio) DNA sequencing techniques allows us to carry out complete characterization of the environmental resistome using DNA obtained directly from environmental samples. Metagenomics enables a comprehensive analysis of the resistome, identifying novel ARGs and assessing their genetic context (e.g., plasmids, transposons), but it involves higher costs and greater bioinformatics complexity [57,58,59,60,61,62].

To achieve effective control of AMR in aquatic environments, it is necessary to implement solid public policies and strict regulations on the use of antibiotics in aquaculture, limiting their use to cases supported by veterinary diagnosis. At the same time, the adoption of good waste management practices and the proper treatment of effluents from aquaculture units are essential to prevent the spread of AGRs in the environment and to preserve the efficacy of antibiotics, thus protecting human, animal and environmental health. In 2015, WHO launched the Global Antimicrobial Resistance Surveillance System (GLASS), which progressively integrates data on antimicrobial use and resistance in humans, animals, and the environment, providing a standardized approach to collect, analyze, and share data, filling knowledge gaps and supporting strategies at multiple levels [37,63,64].

## 7. Conclusions

AMR in aquatic environments represents a growing threat to public, animal and environmental health. The presence of ARGs in aquatic resistomes favors their proliferation and dissipation into pathogenic microorganisms, especially due to anthropogenic pressures such as intensive aquaculture, indiscriminate use of antibiotics and inadequate effluent discharge. This article highlights the importance of HGT mechanisms in the dissemination of ARGs through MGEs such as plasmids, transposons and integrons.

The diversity of bacterial reservoirs, including commensal communities and cyanobacteria, associated with the formation of mixed biofilms and the presence of eDNA creates favorable conditions for genetic exchange and increased resistance. These dynamics aggravate the risk of multidrug-resistant strains emerging in aquatic ecosystems, with direct impacts on human health and ecological balances.

The One Health concept is essential to understanding and combating this problem, promoting the implementation of effective public policies and strict regulations on the use of antibiotics in aquaculture, and the use of monitoring tools such as eDNA and metagenomics allows for the early detection of ARGs and monitoring of AMR in aquatic environments, contributing to the protection of global health.

## Figures and Tables

**Table 1:** Comparison between native resistome and acquired resistome.

Key Features	Native Resistome	Acquired Resistome
Definition	Set of ARGs present in the bacterial genome that evolved naturally over millions of years in natural communities [9,10]	Set of ARGs recently acquired by bacteria in response to anthropogenic pressures, mainly due to the intensive and unregulated use of antibiotics in medicine, agriculture and, particularly, in aquaculture [9,11].
Habitat	Associated with natural ecosystems (soils) and environments with low anthropic influence (deep marine sediments or remote marine areas) [11,12].	Associated with environments affected by human activity, such as aquatic systems exposed to antibiotic residues (wastewater) [10].
Genetic Dynamics	ARGs are integrated into the bacterial chromosome and are not associated with mobile genetic elements. They are rarely involved in horizontal gene transfer [11,12].	ARGs are associated with mobile genetic elements (plasmids, transposons, integrons), which favors their transfer between bacterial species. They are transmitted vertically between generations and horizontally via conjugation, transformation or transduction [9,10,13,14].

**Table 2:** Main genetic elements involved in the dissemination of ARGs in aquatic environments.

Mobile Genetic Element	Description
Plasmids	DNA molecules capable of autonomous replication can often carry multiple ARGs, thus increasing the spread of antibiotic resistance [20,22,23].
Transposons	Mobile DNA segments capable of moving between plasmids and bacterial chromosomes through transposase activity. Transposons often contain ARGs, thus increasing the spread of these genes in bacterial populations [20,23].
Integrons	These are specific genetic elements for the capture and expression of gene cassettes, often including ARGs. Class 1 integrons are the most important in the dissemination of ARGs between bacteria [20,23].

## Data Availability

No new data were created or analyzed in this study. Data sharing is not applicable to this article.
